# Editorial: Women in plant nutrition: 2022

**DOI:** 10.3389/fpls.2023.1271399

**Published:** 2023-09-05

**Authors:** Stefania Astolfi, Petra Bauer, Tzyy-Jen Chiou, Bahar Yildiz Kutman

**Affiliations:** ^1^ Department of Agriculture and Forest Sciences (DAFNE), University of Tuscia, Viterbo, Italy; ^2^ Institute of Botany, Heinrich Heine University, Düsseldorf, Germany; ^3^ Cluster of Excellence on Plant Sciences (CEPLAS), Heinrich-Heine University, Düsseldorf, Germany; ^4^ Agricultural Biotechnology Research Center, Academia Sinica, Taipei, Taiwan; ^5^ Institute of Biotechnology, Gebze Technical University, Gebze, Kocaeli, Türkiye

**Keywords:** plant nutrition, iron, phosphorus, nitrogen, seed

Based on the UNESCO Institute for Statistics data (UIS—UNESCO Institute for Statistics, 2020), female scientists are a minority among researchers and less than 30% of researchers worldwide are women. For sustainable development, science and gender equality are critical concepts. To promote gender equality and highlight the significant contributions of women, Frontiers in Plant Science offered “Women in Plant Nutrition: 2022” platform to promote the work of women scientists particularly in all fields of Plant Nutrition.

The yield and quality of crop plants depend on the abilities of plants to obtain and utilize essential nutrients for their growth and development. Two vital macronutrients for plants are nitrogen and phosphorus. A crucial micronutrient is iron. Understanding the nutritional properties of these elements and how plants acquire them or react to deficiencies is essential for optimizing plant growth and ensuring sustainable agriculture practices. By understanding the mobilization and allocation of nitrogen, phosphorus, and other essential nutrients, we can cultivate healthier crops, protect the environment, and meet the increasing demands for food.

Nitrogen (N) nutrition in plants has significant implications for their growth, development, and overall productivity. The management of N nutrition requires to find a balance between providing adequate N for healthy growth and avoiding overuse that could harm the environment and alter ecological dynamics. Soil management, proper fertilization practices, and sustainable agricultural techniques can help achieve this balance while optimizing plant productivity and preserving environmental and public health. For improving the N fertilizer economy and sustainable agriculture, Padhan et al. attempt to gain an understanding of the processes and mechanisms associated with reproductive stage N remobilization and N partitioning to grain. On the other hand, Ji et al. proposed a sensor-based N-management strategy (GreenSeeker sensor) for the development and implementation of vegetable N-management strategies.

Being able to detect and protect plants from deficient phosphorous (P) is very relevant. P is taken up in the form of phosphate, that is highly relevant as macronutrient. However, phosphate stores are limited and have been only found in few countries. Knowing how phosphate can be better acquired, mobilized, reused or recycled is a topic in several articles of this Research Topic. To increase the availability of insoluble phosphorus for plant use, Yahya et al. study the phosphate-solubilizing bacteria (PSB) by integrating soil nutritional status and meteorological conditions at the application site for sustainable wheat production. These PSB can be developed into potential biofertilizers in the future. Another mechanism by which plants achieve access to phosphate is the onset of autophagy, a controlled degradation and recycling pathway to mobilize the cells’ own nutrient resources under stress and senescence. Lin et al. investigate the roles of two phosphate deficiency-induced autophagy-related proteins.

The study of Le Tougaard et al. aimed at demonstrating how phosphate deficiency can be better diagnosed in leaves in the field, especially when varying light conditions influence the symptoms differently in the various leaf developmental stages. As phytic acid (PA), the major phosphorus storage sink within the plant seeds, binds important essential minerals limiting their bioavailability, Fritelli et al. showed a genetic approach (TILLING strategy) to silence *TdMRP3* and improve micronutrients (Fe, Zn, Mn) concentration in wheat seeds through a significant reduction in PA content.

In addition to absorbing mineral nutrients directly by plant roots, most plant species can acquire nutrients such as phosphate from arbuscular mycorrhizal (AM) fungi *via* symbiotic interaction in natural conditions. The extraradical hyphal network of AM fungi facilitates the transport of nutrients, especially phosphorus, to the plant host in exchange for carbohydrates. Deng et al. characterized the nuclear factor Y (NF-Y) family members in Medicago. They found these transcription factors play crucial roles in arbuscular development and degeneration, offering new insights into the transcription program of AM symbiosis. Hsieh et al. took another aspect to investigate how AM symbiosis affects rice growth and ion homeostasis under salt stress. Their results suggest the potential interplay between phosphate-related signaling pathways and AM-enhanced salt tolerance.

Finally, seed nutritional properties are important for nutritional food security and selecting variants with the highest bio-available nutrient profiles is crucial to meet the global food demands. Iron is an essential micronutrient that frequently limits plant growth under alkaline and calcareous growth conditions. Iron is also frequently lacking in sufficient amounts in human diets. Knowing the genes impacting the uptake and allocation of iron in seeds is a milestone in improving plant-based nutrition. Lichtblau et al. describe a potential regulatory module for iron utilization and accumulation in seeds. Manipulating the amino acid residues involved in the interaction of transcription factors and regulatory proteins (including a small protein and putative E3 ligase) that can form a protein interaction complex is a novel suggested way for biofortification and coping with low Fe bioavailability. Dragicevic et al. found that nutrient properties of maize kernels depended on various kernel traits such as kernel color.

Taken together, this Research Topic highlights mechanisms for nitrogen and phosphate use, as well as potentially new mechanisms for increasing nutritional properties of seeds and kernels. These topics are important for improving nutritional food security and contributing to a better sustainable agriculatural land use system.

## Author contributions

SA: Writing – original draft, Writing – review & editing. PB: Writing – original draft, Writing – review & editing. T-JC: Writing – original draft, Writing – review & editing. BK: Writing – original draft, Writing – review & editing.

